# Synergistic effects of bee venom, hesperidin, and piperine with tamoxifen on apoptotic and angiogenesis biomarker molecules against xerographic MCF-7 injected rats

**DOI:** 10.1038/s41598-023-50729-6

**Published:** 2024-01-17

**Authors:** Abeer A. Khamis, Ehab M. M. Ali, Elsayed I. Salim, Mohamed A. Abd El-Moneim

**Affiliations:** 1 Biochemistry Division, Chemistry Department, Faculty of Science, Tanta University, Tanta, 31527 Egypt; 2 Department of Biochemistry, Faculty of Science, King Abdulaziz University, 21589 Jeddah, Saudi Arabia; 3 Chemistry Department, Faculty of Science, Tanta University, Tanta, 31527 Egypt; 4 Zoology Department, Faculty of Science, Tanta University, Tanta, Egypt; 5 Biochemistry Department, Faculty of Dentistry, Sinai University, Al-Arish, North Sinai Egypt

**Keywords:** Biochemistry, Biological techniques, Cancer, Cell biology, Chemical biology, Drug discovery, Diseases

## Abstract

Breast cancer ranks as the second leading most significant of mortality for women. Studies have demonstrated the potential benefits of natural compounds in cancer treatment and prevention, either in isolation or in conjunction with chemotherapy. In order to improve Tamoxifen's therapeutic efficacy in in-vivo studies, our research sought to determine the effects of hesperidin, piperine, and bee venom as natural compounds, as well as their combination effect with or without Tamoxifen. First, 132 female albino rats were equally divided into six groups and five subgroups, and breast cancer was induced in the selected groups by xenografting of MCF7 cells. Second, the effect of single and best ratio combinations treatment from previous in vitro studies were selected. Next, tumorous mammary glands were collected for apoptotic and antiapoptotic biomarkers and cell cycle analysis. Single or combined natural products with or without Tamoxifen revealed a significant up-regulation in apoptotic genes Bax and Casp3 and a downregulation of antiapoptotic and angiogenesis genes Bcl-2 and VEGF genes. We found that cell cycle arrest in the G0/G1 phase was exclusively caused by Tamoxifen and/ or hesperidin. However, the cell cycle arrest in the G2/M phase is a result of the combination of piperine and bee venom, with or without Tamoxifen by using the flow cytometric technique. Our research concludes that bee venom, hesperidin, and piperine can synergistically enhance to increase Tamoxifen's efficiency in the management of breast cancer.

## Introduction

Breast cancer is considered the most common cancer in women globally and the primary cause of cancer-related deaths in this population. This tumor can spread to other parts of the body but generally begins in the inner lining of lobules or milk ducts^1,2^. Many forms of breast cancer exist, such as ductal and lobular carcinomas, which represent an important percentage of cancers in women^3^. The most prevalent cancer among Egyptian women is breast cancer (16.4%), which is followed in frequency by non-Hodgkin lymphoma (5.4%), bladder cancer (7.9%), and lung cancer (4.9%)^4^. Radiation therapy, chemotherapy, immunotherapy, surgery, and hormone therapy are among potential possibilities for treatment. Even with advancements in cancer treatment, over 80% of patients still face significant problems from side effects and drug resistance^4,5^. Tamoxifen (TMX) is a commonly prescribed medication for the treatment of estrogen receptor-positive breast cancer. TMX has also been proposed as a long-term therapeutic option for patients with premenstrual breast cancer^6^. Ovarian, endometrial hyperplasia, and carcinomas, as well as vein thrombosis and pulmonary embolism, are all serious side effects of such a lengthy treatment^7,8^. Thus, chemosensitization of tumor cells to conventional treatment using non-toxic natural chemicals is a new and unique strategy. These natural compounds are aimed at increasing the cytotoxic efficiency of anticancer drugs, limiting their harmful side effects, and delaying the emergence of acquired chemoresistance^9^. Free radicals have a well-established involvement in the beginning of different disorders. Plant-based antioxidants may diminish the effects of free radicals, hence reducing oxidative damage. This leads to an increase in defense against illnesses including cancer and coronary heart disease^10,11^.

Natural compounds such as bee venom (BV), hesperidin (Hes), and piperine (Pip) were shown to exhibit anti-cancer properties when applied in isolation or in conjunction with anti-cancer medications^5,12^. Flavonoids like Hes was found in significant amounts in a variety of vegetables and fruits^13,14^. It is an affordable byproduct that contains essential bioflavonoids found in citrus fruits like lemon and orange^15^. Hes has a variety of health benefits, including anti-allergic, anti-oxidant, and anti-inflammatory properties^16^. Also, it has anticarcinogenic effects in the tongue, esophagus, colon, and urinary bladder in rat carcinogenesis models^17^.

Pip plant, usually referred to as black pepper, produces piperine, a common alkaloid, primarily in its fruits and roots^18^. Piperine's anti-inflammatory, immunosuppressive, and antibacterial activities have all been studied extensively. Furthermore, several additional studies have demonstrated the chemopreventive or anti-cancer properties of Pip^19–22^. While, rheumatoid arthritis and other conditions have long benefited from the anti-inflammatory properties of bee venom, which is also utilized for relieving pain. Phospholipase A2 (PLA2) and melittin are the two main compounds found in BV. Melittin the major active component it has demonstrated anti-tumor and anti-apoptotic properties^23^. Our previous study used TMX to examine the possible synergistic anti-cancer effects of these three natural ingredients in vitro^5^. This work was carried out to investigate this effect in an in vivo model, and we expected that this combination would have a synergistic effect. As a result, we looked at the effects of these natural chemicals on (ER) positive breast cancer in rats when they were used alone or in combination with TMX.

## Materials and methods

### Chemical and reagents

Hes and Pip (Acros Organics, USA; Cat no. 123460050, 381450050). Bee venom (Apis Injeel ™, Heel GMBH, Germany). TMX (Nolvadex^®^, Astra Zenca Cambridge, UK), MTT [3-(4,5-Dimethylthiazol-2-yl)-2,5-diphenyltetrazolium bromide, Molecular probes, Eugene, Oregon, USA; Cat.no.V-13154)]. Dulbecco's Modified Eagle's Medium (DMEM) (GIBCO, New York, USA; Cat.no.11995073). Fetal bovine serum (GIBCO, Grand Island, New York, USA; Cat.no.10099133). Penicillin/streptomycin (Thermo Fisher Scientific; Waltham, MA, USA; Cat.no. SV30082). l-gluTMXine (Invitrogen, Grand Island, New York, USA; Cat.no. 25030024). Phosphate buffer saline PBS (pH 7.4). Trypsin-EDTA (Invitrogen, Mount Waverly, VIC, Australia; Cat.no. 25200056). DMSO (Sigma Aldrich, St. Louis, MO, USA; Cat no.673439).

### Xerographic MCF-7 injected rats model

132 female albino rats aged (4–6) weeks and weighed approximately (150–170 g), were purchased from the National Cancer Institute (Cairo University, Egypt). The rats were housed at optimum temperature (23–25 °C, relative humidity 55%) with free access to drinking water and food, and adapted to laboratory conditions for 2 weeks before experimentations^24^. Cyclophosphamide (CTX) was injected intraperitoneally into rats at a dose of (50 mg/kg body weight) for four consecutive days to suppress their immune systems^25^. Human breast MCF-7 (#ATCC HTB-22) cells, have been obtained by the Center of Excellence for Research in Regenerative Medicine, and its Applications, (CERMA) Alexandria University, Egypt from the American Type Culture Collection (ATCC) organization. These cells were cultured in DMEM medium which was supplemented with 10% (v/v) fetal bovine serum (FBS) and 1% penicillin–streptomycin at 37 °C in humidified air with 5% CO_2_ cells. Later, after counting, a volume of 1 ml containing 1 × 10^7^ MCF-7 cells was injected around the teat of the mammary gland intradermally. To initiate tumor formation, rats were given intramuscular estrogen injections (0.01 ml/kg body weight, Folon 5 mg/ml) twice a day before receiving the MCF-7 injection, which typically took about four weeks^16,26^.

### Experimental design

The study protocols were according to the Faculty of Science's Institutional Animal Care and Use Committee's recommendations, Tanta University, Egypt with an ethical approval number (#IACUC-Sci-Tu-0404) also, the study is reported in accordance with ARRIVE guidelines (https://drive.google.com/file/d/1E0TZPW_qFnWZ_HM9jKhIY4q0h0aNYTxL/view?usp=sharing). The rats were equally divided into (6 groups and 5 sub-groups) 12 per group and subgroup as summarized in (Table 1):

**Table 1:** Summary of rats groups.

Groups	Dose/administration	References
Group I: (normal control group)	(1ml 0.9%) saline orally plus injected with (0.5 ml) saline intraperitoneally	
Group II: (breast cancer control group)	(1 × 10^7^ MCF-7 cells/1 ml) around the teat of the mammary gland intradermally	^16^
Group III: (TMX group)	(5mg/kg. body wt) once daily for 4 weeks orally	^27^
Group IV: (treatment groups) tumor bearing rats were treated with BV, Hes, and Pip for 4 weeks and divided into 3 sub-groups	Sub-group IVA: (200 mg/kg body wt.) Hes once daily orally	^28^
	Sub-group IVB: (50 mg/kg body wt.) Pip once daily orally	^29^
	Sub group IVC: (0.5 mg/kg body wt.) BV once daily orally	^30,31^
Group V and group VI: cancer bearing rats were treated with nonconstant combination (comusyn software ) of BV, Hes, and Pip alone or in combination with TMX with the best selected ratio from in vitro study Group V: rats were divided into 2 sub-groups	Sub-group VA: Hes and Pip with a ratio of 1:2 w/v, respectively	^5^
	Sub-group VB: BV, Hes, and Pip with a ratio of 1:2:1 w/v, respectively	
Group VI: rats were divided into 3 sub-groups	Sub group VIA: TMX and Pip with a ratio of 1:4 w/v, respectively	^5^
	Sub-group VIB: TMX, BV, and Pip with a ratio of 1:2:1 w/v, respectively	
	Sub-group VIC: TMX, BV, Hes, and Pip, with a ratio of 1:1:4:1 w/v, respectively	

At the end of the experiment, rats were euthanized by cervical dislocation under sodium pentobarbital anesthesia (300 mg/kg body wt) intraperitoneally^32^, The mammary glands were then extracted sterilely, snap-frozen in liquid nitrogen, and stored at – 80 °C. Using previously published techniques. we homogenized and removed tumor tissue for molecular analysis from the treated rats’ mammary glands, while other fresh sample sections were subjected to flow cytometric analysis^33,34^.

### Analysis of gene expression by quantitative real-time PCR (qPCR)

Using the Gene JET RNA Purification Kit (Thermo Scientific, USA), total RNA was extracted from frozen mammary gland tissue of rats after grinding. To assess RNA amount and purity, a Nanodrop (Q5000, Quawell, USA) was employed. RNA (5 μg) was reverse transcribed using Quantiscript reverse transcriptase. Using the Step One Plus real-time PCR equipment (Applied Biosystems, USA) and specific primers, the relative expression of the BCL2-Associated X Protein (Bax), B-cell lymphoma 2 (Bcl-2), cysteine-aspartic proteases (Caspase 3), and Vascular endothelial growth factor (VEGF) genes. Also, β -actin was used as an internal control were determined (Table 2). The thermal cycling conditions, melting curve temperatures, and relative expression computation using 2^−ΔΔCt^ were all carried out as before explained^5,35^.

**Table 2:** The sequences of forward and reverse primers for candidate genes in the mammary gland of rats.

Gene	Forward primer	Reverse primer	Size (bp)	Accession no.
Bax	ACACCTGAGCTGACCTTG	AGCCCATGATGGTTCTGATC	192	NM_017059.2
Caspase3	GGTATTGAGACAGACAGTGG	CATGGGATCTGTTTCTTTGC	130	NM_012922.2
Bcl2	AGTACCTGAACCGGCATCTG	CATGCTGGGGCCATATAGTT	83	L14680.1
VEGF	GATCATGCGGATCAAACCTCACC	CCTCCGGACCCAAAGTGCTC	209	NM_001287108.1
β-actin	AAGTCCCTCACCCTCCCAAAAG	AAGCAATGCTGTCACCTTCCC	149	NM_031144.3

### Analysis of cell cycle by propidium iodide using flow cytometer

Using a flow cytometer and propidium iodide, we examined the cell cycle. Initially, we used 5 mL of 0.1% collagenase enzyme (Invitrogen, USA) for two hours to separate the tissues of the mammary glands. Next, we used a 50 mL Falcon tube with a 0.7 mm nylon mesh to filter the samples. The tubes were twice cleaned, centrifuged for five minutes at 4500 rpm, and then resuspended in PBS. After that, the cells were preserved in ice-cold ethanol and kept at – 20 °C for a whole day. The cells were resuspended in a propidium iodide (PI) solution containing 100 μl (0.02 mg/mL) PI, 0.1% v/v Triton X-100 in PBS, and 50 μl (0.2 mg/mL) RNase A, then washed twice with PBS and incubated for 30 to 60 min in dark under ambient temperature. The samples were examined using a standardize flow cytometer (Applied Biosystem, USA). We assessed the forward (FS) and side (SS) scatter in order to identify individual cells^36^.

### Statistical analysis

We conducted statistical analysis using one-way analysis of variance (ANOVA) along with Duncan's multiple range test (DMRT). We set the significance level as p < 0.05 in order to calculate mean differences. The obtained results were presented as mean ± SE. We used InStat and Graph Pad software programs to analyze the data.

### Ethics approval

This study was conducted according to the ethical protocols of the Tanta University Faculty of Science's Research Ethical Committee (#IACUC-Sci-Tu-0404), also, the study is reported in accordance with ARRIVE guidelines. (https://drive.google.com/file/d/1E0TZPW_qFnWZ_HM9jKhIY4q0h0aNYTxL/view?usp=sharing).

### Consent for publication

The authors declare that they have agreed to the publication of this work.

## Results

### Assessment of Bax, Casp3, Bcl-2, and VEGF genes expression

**Figure 1:**
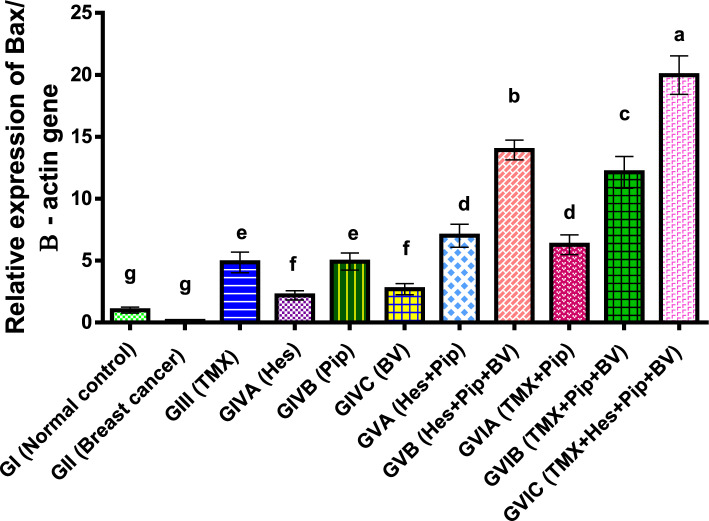
Fold change of Bax gene expression in the mammary gland of tumor bearing rats and treated with TMX alone and combined with BV, Hes, and Pip. a, b, c, d, e, f, and g = *P* < 0.001 if compared with each other groups except (GIIIα GVIA and GIVBα GVIA) = *P* < 0.01 and (GIα GIVA) = P < 0.05.

The results of our investigation displayed that, in comparison to normal control and other treated groups, the expression of Bax was significantly (*P* < 0.01) lower in breast cancer rats. Bax expression levels were, however, significantly elevated by the administration of TMX, BV, Hes, and Pip either singly or in combination, with the groups receiving all four treatment compounds showing the highest levels. TMX and Pip showed noticeably higher expression levels than BV and Hes among the individual treatments. As shown in (Table 3, Fig. 1).

**Table 3:** Effect of TMX, BV, Hes, and Pip on the Bax gene expression in the tumorous mammary gland of rats.

Groups	Fold change mean	± SEM
GI (normal control)	1.00^g^	0.07
GII (breast cancer)	0.13^g^	0.001
GIII (TMX)	4.86^e^	0.24
GIVA (Hes)	2.19^f^	0.11
GIVB (Pip)	4.92^e^	0.2
GIVC (BV)	2.69^f^	0.13
GVA (Hes + Pip)	7.01^d^	0.27
GVB (Hes + Pip + BV)	13.93^b^	0.32
GVIA (TMX + Pip)	6.28^d^	0.23
GVIB (TMX + Pip + BV)	12.13^c^	0.37
GVIC (TMX + Hes + Pip + BV)	19.97^a^	0.45

Means within the same column carrying different superscript letters are significantly different (*P* < 0.05) based on Duncan's multiple range test (DMRT).

**Figure 2:**
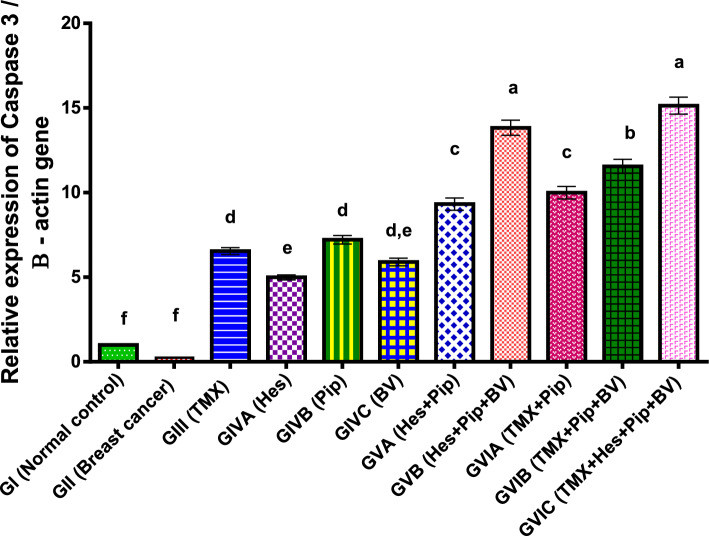
Fold change of Caspase 3 gene expression in the mammary gland of tumor bearing rats and treated with TMX alone and combined with BV, Hes, and Pip. a, b, c, d, e, f, = *P* < 0.001 if compared with each other groups except (GIIIα GIVA and GVIAα GVIB) = *P* < 0.05.

Furthermore, in comparison to normal control and other groups, our study demonstrated a significantly reduced (*p* < 0.01) in the apoptotic gene Caspase 3 expression in the rats with breast cancer. However, Caspase 3 expression was significantly increased by treatment with TMX, BV, Hes, and Pip either separately or in combination as described in (Table 4, Fig. 2).

**Table 4:** Effect of TMX, BV, Hes, and Pip on the Caspase 3 gene expression in the tumorous mammary gland of rats.

Groups	Fold change mean	± SEM
GI (normal control)	1.00^f^	0.02
GII (breast cancer)	0.24^f^	0.01
GIII (TMX)	6.54^d^	0.21
GIVA (Hes)	4.99^e^	0.15
GIVB (Pip)	7.21^d^	0.25
GIVC (BV)	5.90^d, e^	0.23
GVA (Hes + Pip)	9.32 ^c^	0.36
GVB (Hes + Pip + BV)	13.83^a^	0.45
GVIA (TMX + Pip)	9.99^c^	0.37
GVIB (TMX + Pip + BV)	11.55^b^	0.41
GVIC (TMX + Hes + Pip + BV)	15.14^a^	0.5

Means within the same column carrying different superscript letters are significantly different (*P* < 0.05) based on Duncan's multiple range test (DMRT).

**Figure 3:**
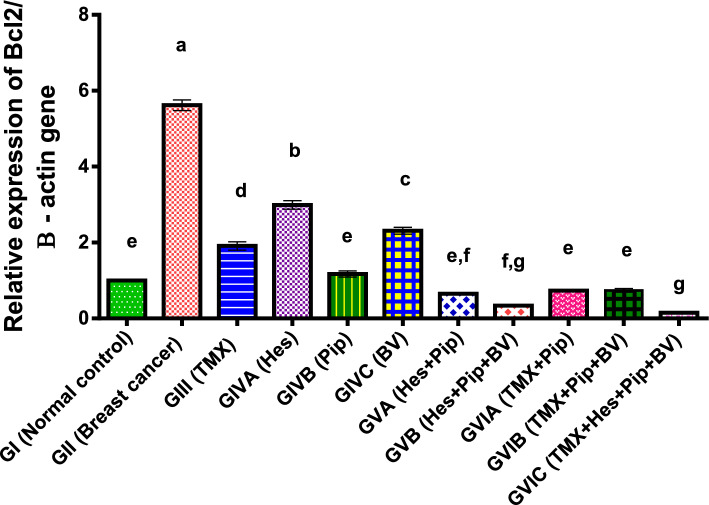
Fold change of Bcl2 gene expression in the mammary gland of tumor bearing rats and treated with TMX alone and combined with BV, Hes, and Pip. a, b, c, d, e, f, g = *P* < 0.001 if compared with each other groups except (GIVBα GVIA and GIVBα GVIB) = P < 0.01 and (GIIIα GIVC, GVBα GVIA and GVBα GVIB) = *P* < 0.05.

In addition, our findings showed that, in comparison to normal control and other treatment groups, the anti-apoptotic gene Bcl-2 expression was significantly higher (*p* < 0.01) in rats bearing breast cancer. The administration of TMX, BV, Hes, and Pip either separately or in combination dramatically decreased Bcl-2 expression. BV and Hes displayed considerably higher expression levels than TMX and Pip among the individual treatments as illustrated in (Table 5, Fig. 3).

**Table 5:** Effect of TMX, BV, Hes, and Pip on the Bcl2 gene expression in the tumorous mammary gland of rats.

Groups	Fold change mean	± SEM
GI (normal control)	1.00^e^	0.02
GII (breast cancer)	5.62^a^	0.14
GIII (TMX)	1.91^d^	0.11
GIVA (Hes)	2.99^b^	0.11
GIVB (Pip)	1.17^e^	0.08
GIVC (BV)	2.31^c^	0.09
GVA (Hes + Pip)	0.65^e,f^	0.01
GVB (Hes + Pip + BV)	0.34^f,g^	0.02
GVIA (TMX + Pip)	0.73^e^	0.02
GVIB (TMX + Pip + BV)	0.74^e^	0.05
GVIC (TMX + Hes + Pip + BV)	0.15^g^	0.01

Means within the same column carrying different superscript letters are significantly different (*P* < 0.05) based on Duncan's multiple range test (DMRT).

**Figure 4:**
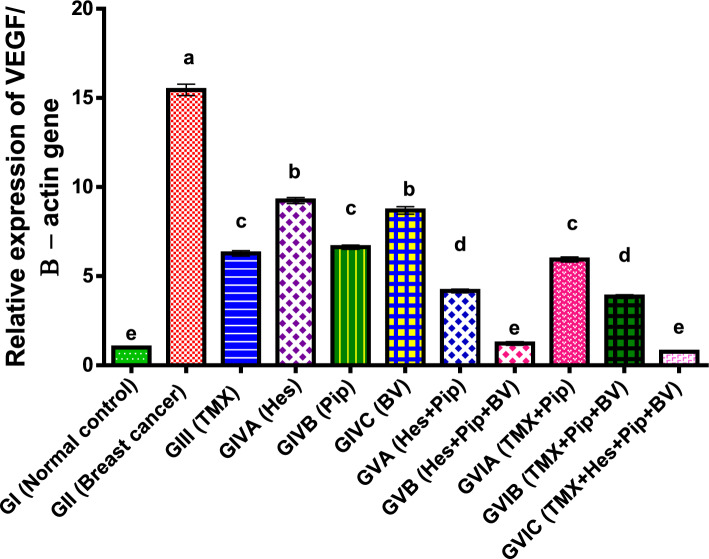
Fold change of VEGF gene expression in the mammary gland of tumor bearing rats and treated with TMX alone and combined with BV, Hes, and Pip. a, b, c, d, and e = *P* < 0.001 if compared with each other groups except (GIVBα GVIA and GIVBα GVIB) = *P* < 0.01 and (GIIIα GIVC, GVBα GVIA and GVBα GVIB) = *P* < 0.05.

Furthermore, in comparison to normal control groups, our experimental results demonstrated a significant (*p* < 0.01) reduced in the angiogenic gene VEGF expression in rats bearing breast cancer. VEGF expression levels significantly decreased in the combination groups, particularly in the groups that included all four medications; the combination groups also showed the lowest levels of VEGF expression. Among the individual treatments, TMX and Pip showed noticeably lower expression levels than BV and Hes. Compared to the single treatments, the combination groups' expression levels were much lower as shown in (Table 6, Fig. 4).

**Table 6:** Effect of TMX, BV, Hes, and Pip on the VEGF gene expression in the tumorous mammary gland of rats.

Groups	Fold change mean	± SEM
GI (normal control)	1.00^e^	0.02
GII (breast cancer)	15.45^a^	0.32
GIII (TMX)	6.28^c^	0.15
GIVA (Hes)	9.25^b^	0.17
GIVB (Pip)	6.63^c^	0.12
GIVC (BV)	8.69^b^	0.21
GVA (Hes + Pip)	4.17^d^	0.11
GVB (Hes + Pip + BV)	1.23^e^	0.1
GVIA (TMX + Pip)	5.94^c^	0.14
GVIB (TMX + Pip + BV)	3.86^d^	0.14
GVIC (TMX + Hes + Pip + BV)	0.76^e^	0.07

Means within the same column carrying different superscript letters are significantly different (*P* < 0.05) based on Duncan's multiple range test (DMRT).

### Examination of the cell cycle in rat mammary tissues: normal and tumorous

**Figure 5:**
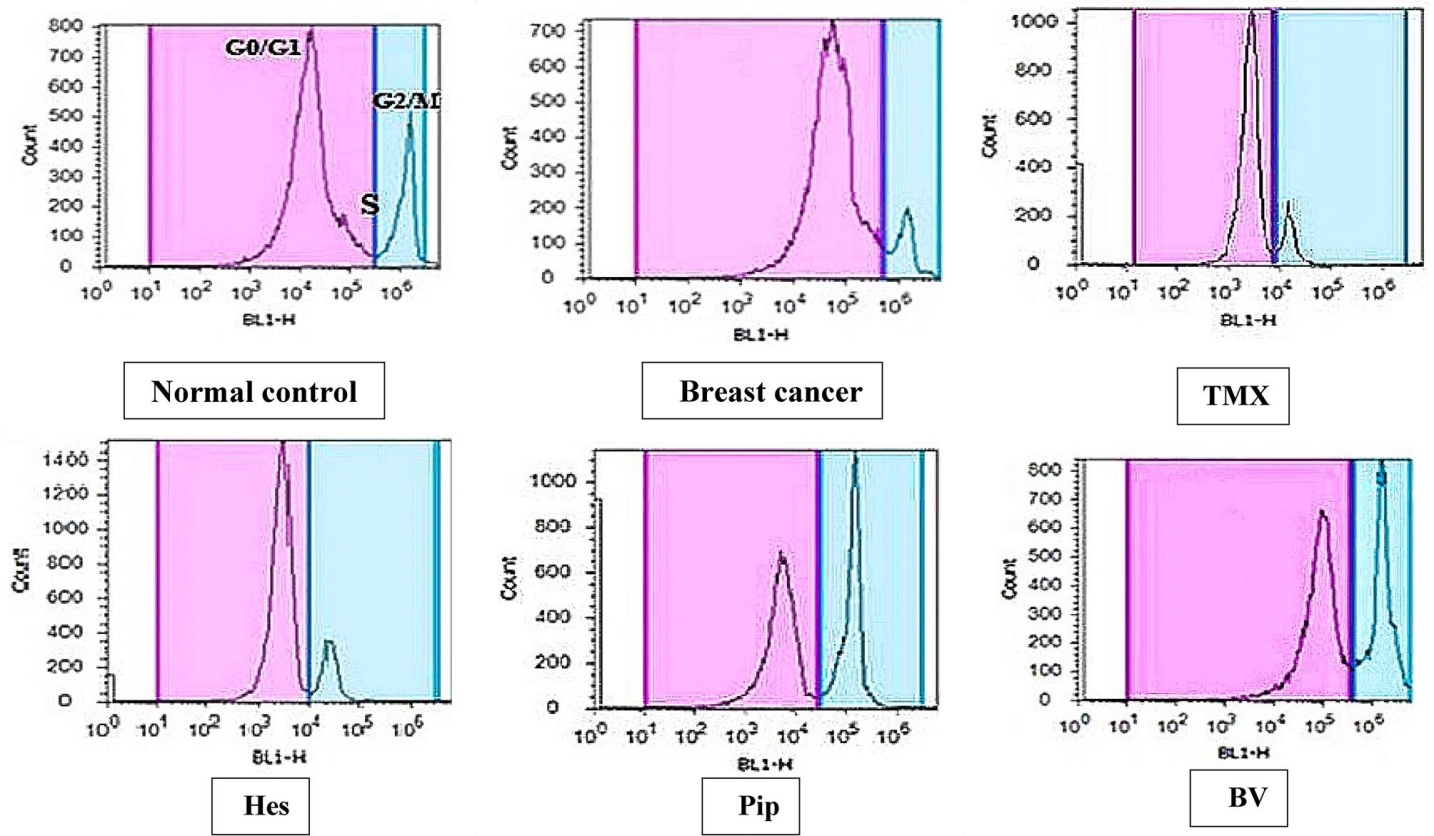
The impact of TMX, BV, Hes, and Pip therapy on the cell cycle of both tumorous and normal mammary glands in rats. The PI fluorescence based on DNA content is represented on the X-axis, while the cells number in each phase is on the Y-axis.

**Figure 6:**
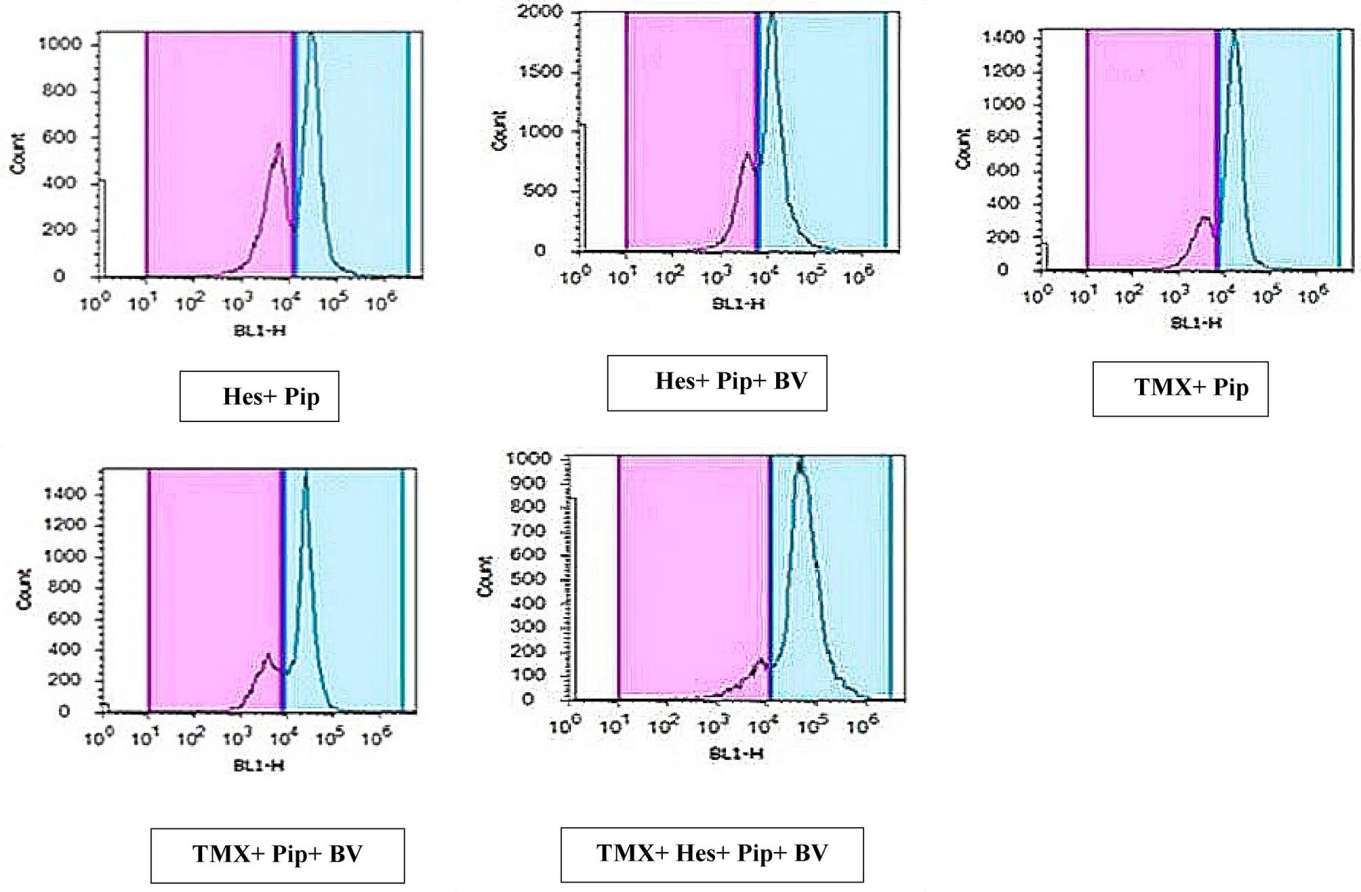
The impact of TMX combined with BV, Hes, and Pip therapy on the cell cycle of both tumorous and normal mammary glands in rats. The PI fluorescence based on DNA content is represented on the X-axis, while the cells number in each phase is on the Y-axis.

**Figure 7:**
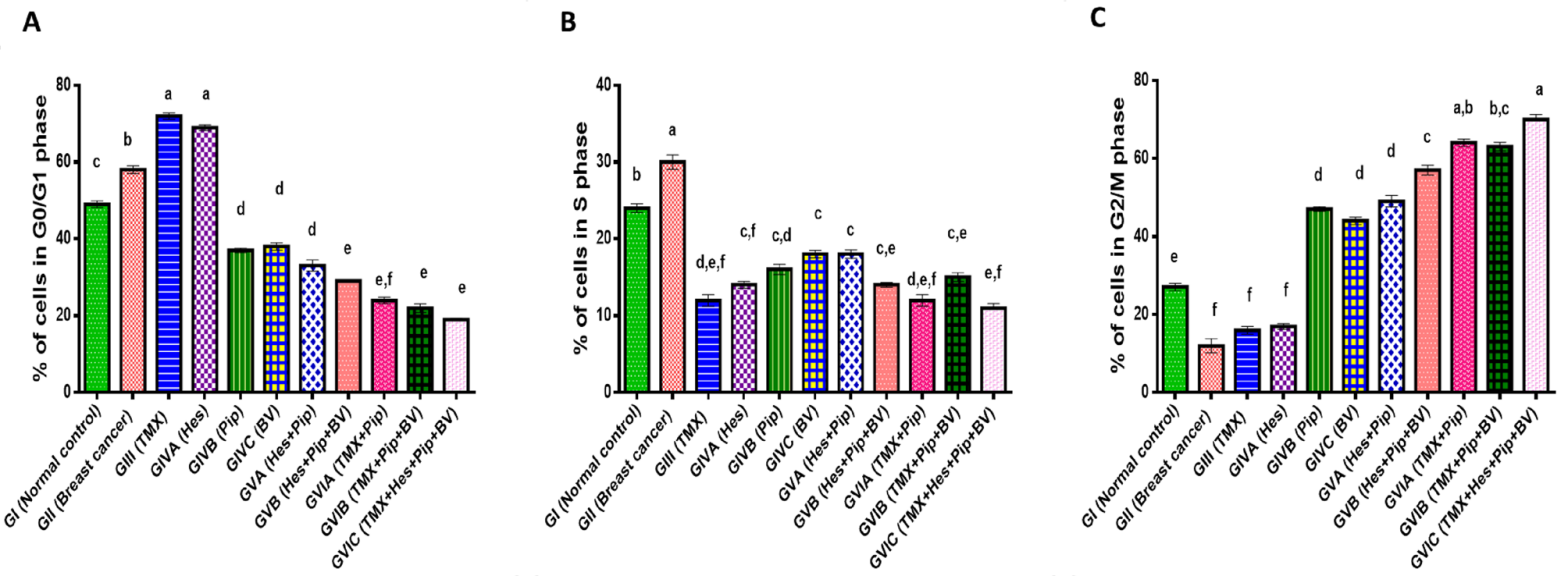
The percentage of cells in the cycle phases in the (**A**) G0/G1, (**B**) S, and (**C**) G2/M phases of the cell cycle in both normal and malignant mammary glands is represented graphically values are calculated as mean ± SEM, n = 12. Means within columns with different superscript letters (a, b, c, d, e, f, and g) are significantly different (*P* ≤ 0.01) if compared with each other groups.

We assessed the effects of individual or combined TMX, BV, Hes, and Pip treatments on the cell cycle using flow cytometry. When PI was administered to rat mammary tissues, both tumorous and normal, the tumorous mammary cells number in the G2/M phase increased significantly when compared to untreated xerographic MCF-7 injected rats (XMR) in the BV and Pip single-treated groups and all combinatorial treated groups (excluding the TMX + Hes group) (Table 7, Figs. 5, 6, 7). The group that received all four medications (TMX, BV, Hes, and Pip) had the highest amount of cells in the G2/M phase. On the other hand, the number of tumorous mammary cells in the G0/G1 phase significantly increased in response to treatment with TMX and/or Hes. When compared to untreated XMR, all single and combination treatments dramatically diminished the number of tumorous mammary cells in the S phase.

**Table 7:** Percentage of cells in each cycle phase in rats' normal and tumorous mammary glands.

Groups	% of cells in each cell cycle phase		
	G0/G1 phase	S phase	G2/M phase
GI (normal control)	49%^c^ ± 1.22	24%^b^ ± 1.01	27%^e^ ± 0.95
GII (breast cancer)	58%^b^ ± 1.01	30%^a^ ± 0.75	12%^f^ ± 1.31
GIII (TMX)	72%^a^ ± 2.01	12%^d,e,f^ ± 0.49	16%^f^ ± 0.91
GIVA (Hes)	69%^a^ ± 1.36	14%^c,f^ ± 0.93	17%^f^ ± 0.58
GIVB (Pip)	37%^d^ ± 1.11	16%^c,d^ ± 0.6	47%^d^ ± 1.39
GIVC (BV)	38%^d^ ± 0.99	18%^c^ ± 0.55	44%^d^ ± 1.02
GVA (Hes + Pip)	33%^d^ ± 1.09	18%^c^ ± 0.97	49%^d^ ± 1.27
GVB (Hes + Pip + BV)	29%^e^ ± 0.95	14%^c,e^ ± 0.62	57%^c^ ± 1.51
GVIA (TMX + Pip)	24%^e,f^ ± 1	12%^d,e,f^ ± 1.15	64%^a,b^ ± 1.47
GVIB (TMX + Pip + BV)	22%^e^ ± 1.34	15%^c,e^ ± 1.2	63%^b,c^ ± 1.33
GVIC (TMX + Hes + Pip + BV)	19%^e^ ± 1.08	11%^e,f^ ± 0.82	70%^a^ ± 1.74

Means within the same column carrying different superscript letters are significantly different (*P* < 0.05) based on Duncan's multiple range test (DMRT).

## Discussion

Breast cancer treatment regimens have been devised, but they are insufficient for comprehensive disease care because of side effects, drug resistance, and the lack of specificity of existing medicines^37^. TMX is an essential anticancer medication that targets the ER and has anti-estrogenic and estrogenic qualities. It is utilized in the avoidance and breast cancer treatment. By suppressing antiestrogen and competitively limiting the estrogen required for proliferation, it prevents ER cancer cells from growing^38^. According to Hammarström et al., postmenopausal symptoms such vaginal dryness, night sweats, discharge or irritation, hot flushes, and irregular menstruation are among the most frequent side effects of TMX^39^. Nevertheless, serious side effects like thromboembolism, secondary endometrial cancer, and endometrial polyps and hyperplasia are frequently brought on by this prolonged treatment plan^40^.

The toxicity of chemotherapy medications is the main reason for the rise in the use of herbal and natural treatments for cancer treatment. It is thought that combining natural items with traditional anticancer medications can increase therapy efficacy through possible synergistic or additive effects. Additionally, this combination might lessen the side effects of chemotherapy^41^. Natural anticancer medicines are safe, easily accessible, and have a synergistic therapeutic effect that may lead to lower dosages, reduced toxicity, and decreased drug resistance during chemotherapy, according to Khamis et al. (2018) give support to this notion.

BV, Hes, and Pip are some examples of natural chemical compounds that possess antibacterial, antioxidant, anti-inflammatory, immunomodulatory, and anticancer properties that may help prevent disease. Hes^42^, Pip^43,44^, and BV^23^**.** Although previous investigations have demonstrated that these natural substances can stop breast cancer cells from growing, not one study has examined how TMX works in conjunction with them. The study we performed looked into how these three natural substances affected the growth of breast cancer cells, both on their own and in combined with TMX. All of these substances, either used singly or in combination, dramatically slowed the growth of tumors; the combination of TMX, BV, Hes, and Pip had the greatest impact^5^. We chose the 10 combination and single therapies with the lowest TMX dosages for in vivo testing in a rat model based on our prior investigations^5^.

According to the study, BV, Hes, and Pip are natural compounds that can cause cells to undergo apoptosis whether used alone or in conjunction with the anti-tumor medication TMX. The pro-apoptotic genes Bax and Caspase 3 were found to be more expressed, while the anti-apoptotic gene Bcl-2 was found to be less expressed. Combining the four compounds resulted in the highest expression of Bax and Caspase 3 genes, whereas Bcl-2 expression was lowest, suggesting a higher rate of apoptosis. BV, Hes, and Pip have also been shown in earlier studies to inhibit the growth of ER-positive breast cancer cells which is in line with our findings^7,45,46^. The three natural products also induce apoptosis in a various of cancers both in vitro and in vivo, including prostate cancer^47^, hepatocellular carcinoma^48^, ovarian adenocarcinoma^44^, and pancreatic cancer cells^49^, through downregulation of Bcl-2 expression, and up-regulation of Bax and Caspase 3 mRNA. This shows that these natural substances' anti-tumor activities are not specific to a particular type of cancer cell.

However, considerable disagreement with other studies that proposed TMX caused apoptosis without effect on Bax gene and by through down-regulation of Bcl-2 gene^50,51^. In other studies, TMX at high doses increased the MCF-7 cells proliferation. The levels of Bcl-2 and Bax remained unchanged^52^. This difference in finding may be due to the dose and time-dependent on the experimental setting. However, the combination of TMX and metformin resulted in a downregulation of anti-apoptotic genes expression like Bcl-2 and an upregulation of pro-apoptotic genes expression like Bax and Caspase 3^53^.

Vascular endothelial growth factor (VEGF) plays a crucial role in controlling tumor angiogenesis and the growth of blood vessels in breast cancer^54^. Our data revealed a significant downregulation in the expression of VEGF gene in TMX and the three natural compounds rats groups. These findings are in accordance with previous studies focusing on different types of in vitro and in vivo cancer model including breast cancer^54–56^, lung metastasis^57^, solid Ehrlich carcinoma (SEC) in mice^58^.

The flow cytometry method was used to examine the breast cancer cells. It was found that TMX, BV, Hes, and Pip treatment had considerable cytotoxic effects on cells. Cells were arrested by the compounds at two distinct phases of the cell cycle: the G2/M phase was arrested by BV and Pip, and the G0/G1 phase was arrested by TMX and Hes. The greatest number of arrested cells was produced by the combination of all four chemicals. These findings are consistent with previous research Ip et al.^45,59^ that showed BV and Pip induce G2/M phase arrest in malignant breast cells and G1 phase arrest in a number of different cancer cell types^46^ including prostate cancer^60^. by targeting different cell cycle regulators. On the other hand, our study demonstrated that TMX and/or Hes promoted tumorous mammary cells to enter the G0/G1 phase which has also been observed in other studies^61^. As a result, TMX and Hes have both cytostatic effect by G0/G1 arrest and cytotoxic activity by inducing apoptosis^62^. It's interesting to point out that, all combinations, except TMX + Hes, arrested tumorous mammary cells in the G2/M phase of the cell cycle. Also, our result proved that the highest number of arrested cells in combinatorial groups. This finding confirmed that the four chemicals' strongest impact on the cell cycle arrest phase could be due to their synergistic effect.

## Conclusion

In conclusion, a combination of standard chemotherapy agents like TMX with plant-derived naturally occurring substances like BV, Hes, and Pip has shown a considerable improvement in cancer management against tumorous mammary gland cells. This combination strategy lead to boost efficiency and lower toxicity. Their impact was achieved by the upregulation of apoptotic genes (Bax, Casp3) and the downregulation of antiapoptotic genes Bcl-2 and angiogenesis genes VEGF. In addition, TMX and Hes have both cytostatic effect by G0/G1 arrest and cytotoxic activity by inducing apoptosis. Furthermore, BV and Pip arrested tumorous mammary cells in the G2/M phase of the cell cycle. Finally, natural product combinations are a promising approach for developing more effective and less hazardous cancer treatments than synthetic chemotherapy.

## Data Availability

The authors declare that all relevant data that support the findings of this study are incorporated in the manuscript.

## Abbreviations

TMX: Tamoxifen
ER: Estrogen receptor
BV: Bee venom
Hes: Hesperidin
Pip: Piperine
PLA2: Phospholipase A2
CTX: Cyclophosphamide
DMEM: Dulbecco’s Modified Eagle’s Medium
FBS: Fetal bovine serum
Bax: BCL2-associated X protein
Bcl-2: B-cell lymphoma 2
Caspase 3: Cysteine-aspartic proteases
VEGF: Vascular endothelial growth factor
PI: Propidium iodide
XMR: Xerographic MCF-7 injected rats
